# Sample Size of Trials Investigating the Impact of Point‐of‐Care Ultrasound‐Guided Strategies on Patient Outcomes: A Systematic Review

**DOI:** 10.1002/jum.70001

**Published:** 2025-07-25

**Authors:** William Beaubien‐Souligny, Michel Gouin, Karel Huard

**Affiliations:** ^1^ Division of Nephrology Centre Hospitalier de l'Université de Montréal Montreal Canada; ^2^ Department of Medicine Université de Montréal Montreal Canada

**Keywords:** evidence‐based medicine, point‐of‐care ultrasound, randomized controlled trials

## Abstract

Point‐of‐care ultrasound (POCUS) is increasingly utilized for bedside diagnosis and management in diverse clinical contexts. However, the design of randomized controlled trials (RCTs) evaluating the impact of POCUS‐guided strategies on clinical outcomes presents significant challenges. This study aims to explore the assumptions underlying sample size estimation in POCUS‐guided trials and assess the adequacy of sample sizes in published trials through a systematic review. We performed a sample size analysis considering varying rates of POCUS‐induced management changes and plausible effect sizes on binary and continuous patient‐centered outcomes. Additionally, a systematic review of PubMed was conducted to identify RCTs comparing POCUS‐guided management to usual care, extracting data on planned and actual sample sizes and justifications for sample size decisions. Sample size estimations revealed a substantial dependence on the proportion of participants experiencing management changes due to POCUS findings. For example, achieving adequate power in a trial with a moderate effect size requires over 1000 participants if POCUS alters management in 50% of cases. Our review included 25 RCTs, with a median sample size of 206 participants (interquartile range 122–250). Only 68% of trials reported sample size justifications, and 41% failed to meet planned recruitment targets, primarily due to recruitment challenges and other logistical barriers. Most trials investigating POCUS‐guided strategies are underpowered, underscoring the need for realistic sample size estimations that consider the rate of POCUS‐induced management changes and anticipated effect sizes. Future trials should incorporate pilot phases and innovative designs to optimize feasibility and power.

**Abbreviations**
OR: odds ratio
POCUS: point‐of‐care ultrasound
RCT: randomized clinical trial

Performing a randomized clinical trial (RCT) is well‐established as a necessary step to demonstrate the efficacy of a medical strategy. While the methodology of RCTs has been focused primarily on testing pharmacologic interventions, it is also feasible to investigate other types of interventions including complex management strategies guided by additional information including imaging modalities.^1^, ^2^

It is generally advisable to test new interventions by comparing them to current usual care to determine if the new strategy is truly advantageous. Pharmacology trials imply that the management in the intervention group will be different in almost all participants compared to the usual care group unless non‐adherence or contamination of the control arm occurs at a high rate. In this setting, the difference observed between groups can be inferred to be proportional to the biological effect of the pharmacologic agent. In contrast, in trials involving strategies guided by additional information, the care provided to participants in the intervention group may not necessarily differ from usual care, as other sources of information could lead to the same decision‐making process and result in similar management.

Point‐of‐care ultrasound (POCUS) has seen an increase in popularity in the last decade under the premise that the information provided can significantly impact patient care through early diagnosis or more precise clinical decisions at the beside.^3^ The indications to perform POCUS have progressively widened to include trauma,^4^ respiratory failure,^5^ undifferentiated shock,^6^ acute kidney injury,^7^ and cardiac arrest.^6^ There are numerous testimonies that POCUS can significantly impact the management.^8^ However, despite showing that POCUS examination can hasten the time to the correct diagnosis in a multitude of settings,^9^, ^10^ incertitude remains on how this type of strategy impacts important patient outcomes.^11^

We propose to explore assumptions regarding sample size estimation in trials of POCUS‐guided therapy. We then perform a systematic review of the literature to assess the sample size of published trials and evaluate whether sample size decisions during the design of trials investigating the impact of POCUS‐guided strategies on patient‐centered outcomes are appropriate.

## Materials and Methods

This report includes an analysis of sample size estimations and a systematic review. It does not contain original participant data, and research ethics board approval was therefore not required.

### Analysis Related to Sample Size Requirements

To produce a realistic estimate of sample size requirements for a hypothetical trial with a primary binary patient outcome with 1:1 group attribution, we assumed a rate of 40% in the control group and performed a sample size estimation to achieve a power of 0.8 and a confidence level of 95%. The analysis was repeated for different combinations of the plausible effect size of a POCUS‐guided alteration in clinical management of the studied outcome (odds ratio [OR] 0.6, 07, or 0.8) while considering that these changes would only occur in a given proportion of patients (10%, 25%, 50%, 75%, 100%) which will result in an observed lower adjusted effect size when comparing the intervention and control arms of the trial. The same analysis was repeated using the same methodology but considering a continuous outcome. Analysis was performed in R (version 4.2.2) using package *rpact*. A complete version of the R script is presented in the online supplemental Appendix 1.

### Systematic Review

#### Eligible Studies

We aimed to include completed randomized clinical trials investigating the impact on patient outcome of a management strategy based on information from POCUS compared to usual care. We excluded trials related to the use of POCUS for procedural guidance and for which the primary outcome was not an outcome related to the patient (i.e., diagnostic performance).

#### Search Strategy

We performed a systematic search over the PubMed database in July 2024 combining a validated Cochrane term for randomized clinical trials^12^ and a term specific for studies involving Point‐Of‐Care ultrasound^13^ (see online supplemental Appendix 1). We also identified additional trials through the bibliography of included studies. A first selection was made based on title, with each entry being reviewed by two investigators. A second selection based on abstract and full‐text review was subsequently performed.

#### Data Extraction

We extracted information about the population and the intervention investigated in the selected reports. We collected information about planned and final sample size (*N*), sample size justifications, and reasons for non‐achievement of the planned sample size if reported.

#### Data Analysis

We report the planned and actual sample size of included trials, as well as the relative difference between the final and the planned sample size (as a %). The reasons for non‐achievement of planned sample size are reported in a descriptive manner when available.

#### Risk of Bias and Quality of Evidence

In the selected reports, we determine if the calculation of the required sample size is reported within the text or the associated supplementary material. We did not evaluate other components usually included in the risk of bias assessment due to the specific focus of this systematic review.

## Results

### An Evaluation of Sample Size Requirements

As previously stated, in the context of an RCT evaluating a POCUS‐guided intervention strategy, we can expect that the additional information from POCUS will lead to a change in management in a certain proportion of participants. This proportion may vary depending on the clinical context studied. Since the impact of the POCUS‐guided strategy on patient outcomes can only be mediated through changes in clinical management in response to POCUS findings, we can expect the effective sample size to be much lower than the actual number of participants recruited in the trial.

Another factor to consider is the plausible effect of a POCUS‐guided interventions on patients' outcomes. In some contexts, it can be expected that a relatively rare POCUS finding can lead to a critical change in patient management. For example, finding a significant pericardial effusion during cardiac arrest could lead to a therapeutic pericardiocentesis, which may result in a very large effect on patients' chances of survival. However, in most cases, it is probable that less drastic changes in management will translate to a moderate impact on patients' outcomes. Successful pharmacotherapy trials leading to practice‐changing evidence often demonstrate a moderate effect (OR = 0.7) of an intervention on important patients' outcomes.

According to these considerations, a realistic estimation of sample size requirements in a trial investigating a POCUS‐guided strategy on a binary patient outcome based on the proportion of participants in which POCUS led to a change in management and the expected plausible effect size of these interventions is presented in Table 1.

**Table 1:** Estimated Sample Size Requirements Based on the Proportion of Participants in Whom Ultrasound Led to a Change in Management and on the Plausible Effect Size on a Binary Patient‐Centered Outcome Related to These Interventions

Plausible Effect Size Resulting From POCUS‐Guided Changes in Management (OR)	Proportion of Participants in Which POCUS Results Lead to a Change in Management	Expected Observable Effect Size of the POCUS‐Guided Strategy Compared to Usual Care (OR)	Estimated Sample Size
Large (OR = 0.6)	100%	0.60	208
	75%	0.70	307
	50%	0.80	560
	25%	0.90	1766
	10%	0.96	9389
Moderate (OR = 0.7)	100%	0.70	383
	75%	0.78	593
	50%	0.85	1148
	25%	0.93	3889
	10%	0.97	21,806
Small (OR = 0.8)	100%	0.80	888
	75%	0.85	1440
	50%	0.90	2941
	25%	0.95	10,607
	10%	0.98	62,096

All analysis conducted considering a power of 0.8 and a confidence level of 95% for a dichotomous binary primary outcome occurring at a rate of 40% in the control group in a trial with 1:1 group attribution.

OR, odds ratio; POCUS, point‐of‐care ultrasound.

As an example, if we consider a hypothetical POCUS‐guided strategy that led to a change in management in 50% of participants compared to usual care and that the modification in therapeutic management led to a moderate clinical benefit (OR = 0.70), the expected difference in the incidence of the studied outcome of the POCUS‐guided strategy compared to usual care is expected to be small (OR = 0.85). Consequently, this trial would require a considerable sample size (*N* = 1 148) to achieve sufficient statistical power.

A continuous outcome, such as length of stay in the hospital, could also be used as a patient‐centered endpoint. A similar analysis regarding this type of outcome and the same previous assumptions is presented in Table 2. With a similar example, if POCUS‐guided strategy leads to a change in management in 50% of participants compared to usual care, even if the modification in therapeutic management can lead to a significant reduction in the hospital length of stay (reduction from 10 to 7 days of hospitalization [−3 days, relative 30% reduction]), the expected difference in the incidence of the studied outcome within a trial of POCUS‐guided strategy compared to usual care is expected to be smaller (−1.5 days, relative 15% reduction). Consequently, this trial would require a considerable sample size (*N* = 895) to achieve sufficient statistical power.

**Table 2:** Estimated Sample Size Requirements Based on the Proportion of Participants in Whom Ultrasound Led to a Change in Management and on the Plausible Effect Size on a Continuous Patient‐Centered Outcome Related to These Interventions

Plausible Effect Size Resulting From POCUS‐Guided Changes in Management	Proportion of Participants in Which POCUS Results Lead to a Change in Management	Expected Observable Effect of the POCUS‐Guided Strategy on Continuous Outcome (% Relative Reduction)	Estimated Sample Size
Large (40% reduction in length of stay)	100%	40%	127
	75%	30%	225
	50%	20%	504
	25%	10%	2011
	10%	4%	12,560
Moderate (30% reduction in length of stay)	100%	30%	225
	75%	22.5%	398
	50%	15%	895
	25%	7.5%	3574
	10%	3%	22,328
Small (20% reduction in length of stay)	100%	20%	504
	75%	15%	895
	50%	10%	2011
	25%	5%	8039
	10%	2%	50,234

All analysis conducted considering a parallel RCT with 1:1 allocation with a standard deviation of 80% of the value of the mean for 80% power with 95% confidence.

POCUS, point‐of‐care ultrasound.

### Systematic Review of Planned and Final Sample Sizes in Published Trials of POCUS‐Guided Management

**Figure 1:**
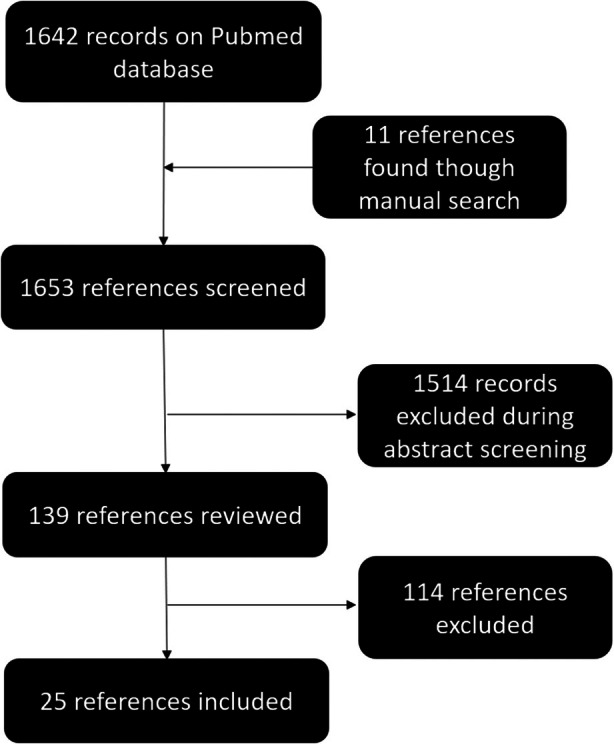
Reference inclusion flowchart.

**Figure 2:**
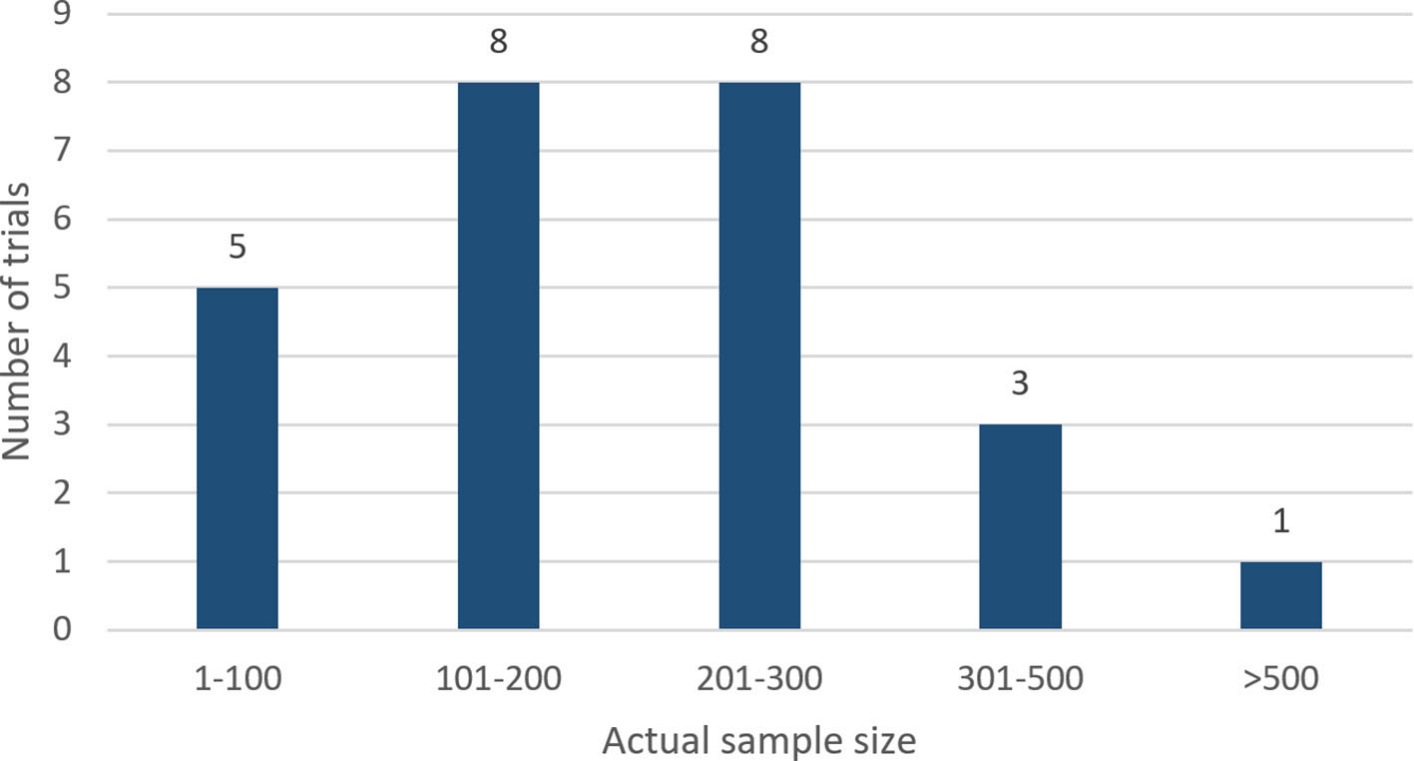
Final sample size of trials comparing POCUS‐guided strategies to usual care for a primary patient outcome (N = 25).

We screened 1653 references and identified 25 eligible trials (Figure 1 and Table 3). The median sample size was 206 participants (interquartile range = 122; 250) as shown in Figure 2. Sample size justifications were provided for 17 trials (68.0%). Among these trials, 7 (41.2%) did not achieve their pre‐specified sample size target with a median relative difference of −31.8% (interquartile range: −26.4; −42.8%) between the planned and final sample size. Several reasons were cited including insufficient recruitment,^14^, ^15^, ^16^ perceived futility,^15^ and other circumstances (pandemic,^17^ site drop‐off^16^).

**Table 3:** Included Studies

Authors/Year	Summary	Primary Outcome	Rate of Change in Management With POCUS	Planned Sample Size	Actual Sample Size	Main Result
Binary primary outcome						
Chardoli et al^24^	POCUS vs usual care during pulseless cardiac arrest	Mortality (ROSC)	NR, potentially addressable findings found in 14%–78% of assessments	NR	100	No difference Rate: 34% vs 28%, *P* = .52
Smith‐Blindman et al^18^	POCUS vs radiology US vs CT for suspected nephrolithiasis	High‐risk diagnosis with complications related to missed or delayed diagnosis	NR	2500	2759 (1:1:1 design)	No difference Rate: 0.7% vs 0.3%, *P* = .30
Siriopol et al^25^	Lung POCUS for fluid management vs usual care in hemodialysis patients	All‐cause mortality and first cardiovascular event—including death, stroke, and myocardial infarction	NR	480	250	No difference HR: 1.09 CI: 0.64; 1.86, *P* = .75
Chen et al^26^	Use of POCUS during morning rounds in patients with sepsis vs usual care	Mortality	17%–29% of encounters	No sample calculation was performed	129 (1:2 design)	No difference Rate: 0.27 vs 0.43, *P* = .11
Atkinson et al^15^	POCUS vs usual care for hypotension in the ED	30‐Day mortality	Not reported, 9%–55% clinically relevant findings	400	273	No difference Rate difference: 0.35% CI: −10.2%, 11.0%
Rivas‐Lasarte et al^17^	Lung POCUS vs usual care during outpatient follow‐up after acute heart failure	Composite of urgent visits, hospitalization for heart failure or death during follow‐up	NR	124	124	Difference HR: 0.52 CI: 0.27, 1.00, *P* = .049
Araiza‐Garaygordobil et al^27^	Lung POCUS vs usual care in outpatients with heart failure	Composite of urgent heart failure visits, re‐hospitalizations or death	NR	124	126	Difference HR: 0.55, CI 0.31–0.98, *P* = .04
Zoccali et al^14^	Lung POCUS for fluid management vs usual care in hemodialysis patients	Composite of all‐cause death, myocardial infarction and decompensated heart failure	NR	500	363	No difference HR: 0.88; CI: 0.63–1.24
Musikatavorn et al^28^	US‐guided fluid management (IVC) vs usual care in adults with sepsis	30‐Day mortality rate	NR	242	211	No difference Rate: 18.8% vs 19.8%, *P* = .84
Li et al^29^	Cardiopulmonary POCUS vs usual care for septic shock	28‐Day mortality	NR	94	94	No difference Rate: 50.6% vs 60.0% *P* = .58
Ricci et al^30^	POCUS vs usual care to guide diuretic adjustments in acute heart failure	30‐Day readmission rate	NR	250	250	No difference 19% vs 15%, *P* = .40
Torres‐Macho et al^31^	Lung POCUS vs usual care after discharge for acute heart failure	Cardiovascular death, readmission, or emergency department or day hospital visit for heart failure	NR	152	79	No difference 26.1% vs 29.7% *P* = .83
Ravetti et al^32^	POCUS vs usual care after high‐risk surgery	Acute kidney injury	NR	178	111	No difference Rate: 27.5% vs 23.3%, *P* = .66
Zisis et al^33^	POCUS‐guided lung decongestion in decompensated heart failure vs usual care	Hospital re‐admission or death	NR	NR	122	No difference OR: 1.36; CI: 0.59–3.1; *P* = .50
Continuous primary outcome						
Lucas et al^34^	POCUS vs standard echocardiography for inpatients with an indication for echocardiography	Length of hospital stay	37% of patients	420	453	No difference 1.7% CI: −12.1, 9.8%
Han et al^35^	POCUS to monitoring lung congestion vs usual care after pediatric cardiac surgery for congenital disease	Duration of mechanical ventilation	NR	NR	100	Difference 15.0 vs 38.5 hours *P* = .04
Park et al^36^	Use of POCUS vs usual care in the context of renal colic in the ED	Length of stay in the ED	NR	NR	103	Difference 89.0 vs 163 minutes *P* < .001
Wilson et al^37^	POCUS vs usual care in patients who require a pelvis ultrasound in the ED	Length of stay in the ED	NR	No sample calculation was performed	194	Difference −120 minutes CI: 66–173
Morgan et al^16^	POCUS vs radiology US in pregnant women with abdominal pain or bleeding	Length of stay in the ED	NR	300	224	No difference −20 minutes CI: −54; 7
Kim et al^38^	POCUS vs usual care for renal colic	Length of stay in the ED	NR	152	152	Difference 172 vs 234 minutes *P* < .001
Guner et al^39^	POCUS‐guided diagnostic strategy vs usual care for chest pain	Length of stay in the ED	NR, POCUS led to findings in 31%	NR	208	Difference 133 vs 215 minutes *P* = .006
Cid‐Serra et al.^40^	Multiorgan POCUS vs usual care for patients admitted for a cardiopulmonary diagnosis	Length of hospital stay	NR	250	250	No difference 113 vs 125 hours *P* = .53
Durgun et al^41^	POCUS diagnostic strategy vs usual care for abdominal pain in the emergency department	Length of stay in the ED	NR, findings in 64%	NR	207	Difference 209 vs 286 minutes *P* = .003
Psalidas et al^42^	POCUS vs usual care to evaluate the success of pleurodeses in patients with malignant pleural effusion	Length of hospital stay	NR	254	313	Difference 2 vs 3 days, *P* < .001
Arvig et al^43^	Repeated cardiopulmonary POCUS vs usual care (only at admission) in adult patients admitted for dyspnea	Reduction of dyspnea score	NR	206	206	Difference −1.66 point; CI: −2.09 to −1.23

ED, emergency department; CI, confidence interval; HR, hazard ratio; NR, not reported; OR, odds ratio; POCUS, point‐of‐care ultrasound.

In terms of results, 2/14 (14.3%) of studies investigating a binary outcome reported a significant difference, while 8/11 (72.7%) of studies investigating a continuous outcome (i.e., length of stay) reported a significant result (*P* = .003).

Based on the analysis presented in the previous section, only one RCT^18^ considering a binary primary outcome reported a sample size with appropriate statistical power to detect the impact of a POCUS‐guided strategy, assuming the additional information would have led to a large clinical impact in approximately half of the patients randomized in the intervention group. Of note, in terms of patient‐centered outcomes, the sample size estimation for this study was centered on non‐inferiority of the POCUS‐guided approach related to the occurrence of a patient‐centered binary event (high‐risk diagnoses in the context of suspected urolithiasis).

## Discussion

In this analysis, we first suggest that a realistic sample size estimation regarding trials of POCUS‐guided management should consider the plausible effect of the change in management resulting from the additional information provided, as well as the proportion of participants in the intervention group for whom POCUS results will lead to a change in management compared to usual care. Secondly, through a systematic review, we propose that most previous trials of POCUS‐guided management were generally underpowered according to these considerations, although a higher proportion of trials investigating continuous outcomes demonstrated significant differences in their primary outcome compared to trials investigating a binary outcome.

The concerns related to misleading results that can arise from trials that are underpowered to detect the plausible effect of an intervention have already been explored in other areas of biomedical research.^19^ However, these concerns are particularly relevant to POCUS research, as there are several barriers to conducting multicenter trials on complex medical interventions.^20^ Integrating POCUS as a primary source of information to guide therapy comes with a specific set of challenges which are compounded when the design of a large multicenter trial is considered. First, there must be enough POCUS operators at participating sites, while ensuring that contamination is avoided. This is challenging to achieve since POCUS operators in this context are likely members of the hospital staff and are likely to use POCUS in their practice. Secondly, POCUS skill and interpretation must be standardized within teams and across sites. Therefore, an onboarding strategy including proper training becomes a crucial preliminary step before site activation. Nonetheless, this approach has been shown to be feasible.^21^ In general, according to these considerations, the human and financial costs required to conduct a definitive trial of a POCUS‐guided strategy are substantial.

Our review suggests that researchers in this field may not have been fully prepared. An important proportion did not meet their target enrollment at the end of the trial, and some authors did not report either the rationale of sample size estimation or the actual enrollment target. The strategies to overcome the barriers associated with the design and conduct of RCTs of POCUS‐guided strategies have seldom been explored. One approach would be to ensure, prior to designing a large‐scale multicenter trial, that a high rate of management changes in response to POCUS information is expected in the studied population. Epidemiological studies and questionnaires in POCUS users, and pilot trials in target populations would help describe the rate of modification and determine whether a large trial is likely to succeed. For example, Table 1 shows that the required sample size in a population with a 25% rate of modification (*N* = 3 889) would be more than 6 times higher than if the rate of modification is 75% (*N* = 593) considering a high‐impact change in management resulting from POCUS. In this regard, studies investigating the impact of the use of POCUS as a reliable method to safely exclude a diagnosis in the emergency department leading to earlier discharge are more likely to impact a higher proportion of patients. This may explain why studies considering a continuous primary outcome, usually length of stay, were more likely to yield a positive result according to our review. Even if most studies reported a modest reduction of emergency department length of stay (<2 hours), this difference can translate into meaningful improvements in efficiency, resulting in a benefit that extends to other patients as well in the form of reduced waiting time or by enabling clinicians to devote greater attention to emergency department patients with complex needs.

**Figure 3:**
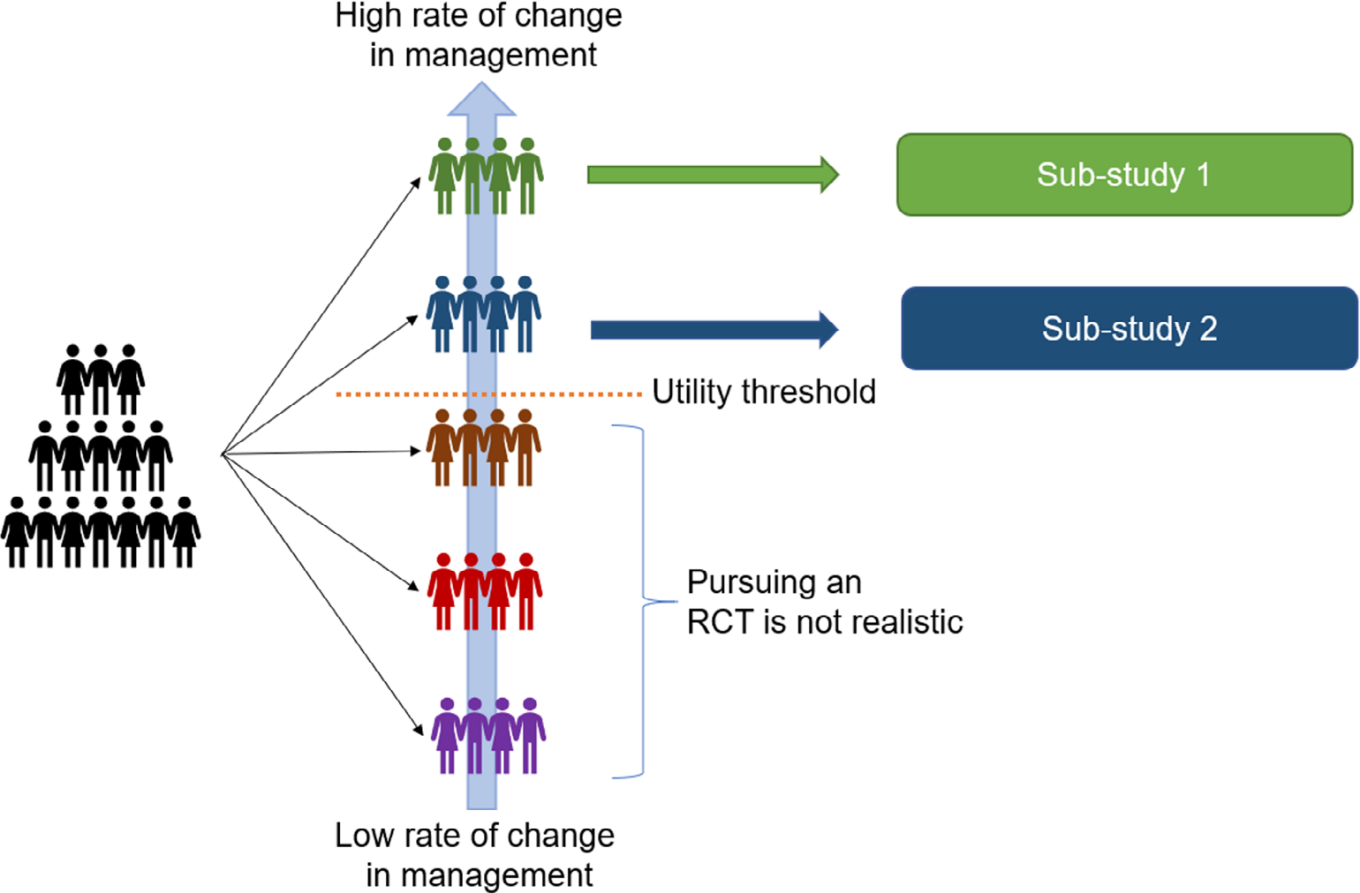
Concept of an adaptive basket trial related to Point‐Of‐Care ultrasound assessment. A large general hospitalized or ICU population is considered at the start of the trial. At a predefined timepoint, the sub‐groups for whom the trial will continue will be selected based on the proportion of participants for whom the information from the POCUS examination results in a change in clinical management.

Despite the potential barriers, performing large‐scale biomarker‐guided trials has been previously done, such as natriuretic peptide‐guided therapy for heart failure, whose benefit is only apparent in large meta‐analysis^22^ despite widespread use in heart failure clinics.^23^ Another approach would be to obtain these key elements directly during a pilot phase of the RCT, which would also enable exploration of optimal workflow and feasibility of the proposed intervention. Enrichment strategies to enroll participants in which POCUS information is the most likely to modify management must be pursued. Novel trial designs, including adaptive basket trials, could in theory be used to define the target subgroups in which the conduct of a large‐scale trial would be feasible without the need for separate pilot trials if the intervention (or type of POCUS assessment) is applicable to this heterogeneous population (Figure 3). Finally, including only participants for whom there is uncertainty related to the use of POCUS according to the treating clinician could also be an important enrichment strategy, although it could lead to significant heterogeneity related to the usual care of practitioners and within institutions.

Our analysis has several limitations. First, this analysis and review excluded use of POCUS for procedural guidance to focus on POCUS‐guided management strategies. Other considerations specific to procedural POCUS could have been relevant to this analysis. Secondly, our sample size estimation is derived from a limited number of possible combinations of parameters. Thirdly, our systematic review was not prospectively registered and is limited to report published in the PubMed database. Finally, we only considered the studies regarding the clinical impact of a POCUS‐guided strategy which exclude studies on other potential benefits of POCUS including diagnostic accuracy. It could be argued that the evidence‐base for the use of the technology could rest entirely upon its benefit, even if the impact on patient outcomes remains elusive. While POCUS has been advocated as the fifth pillar of physical examination, our expectations from an evidence‐based medicine standpoint seems at odds with other traditional components of physical examination. For example, The *Journal of American Medical Association*'s classic series “The Rational Clinical Examination” presents the literature on the diagnostic performance of diverse physical examination maneuvers without regard to whether performing these examinations improves patient outcomes. However, these physical examinations assessments remain widely regarded as the standard for clinicians to determine which skills are worthwhile mastering. Apart from diagnostic accuracy, more appropriate measures of success regarding POCUS may include cost savings in radiology and monitoring apparatus, shorter time to final diagnosis, reduced rate of missed life‐threatening diagnoses, reduced diagnostic uncertainty among clinicians, fewer outpatient visits for additional investigations culminating in greater patient satisfaction.

## Conclusion

The design of RCTs on POCUS‐guided therapy should have realistic considerations regarding sample size requirements to determine the impact on patient outcomes. In theory, this involves producing an accurate estimation of the proportion of participants in which POCUS will yield information that will significantly influence decision‐making. Research efforts should continue to define the optimal approach for generating the evidence base for the use of POCUS in medicine.

## Supporting information


**Data S1.** Supporting Information.

## Data Availability

Data sharing is not applicable to this article as no new data were created or analyzed in this study.
