# Supplementary material for: Sample Size of Trials Investigating the Impact of Point‐of‐Care Ultrasound‐Guided Strategies on Patient Outcomes: A Systematic Review
Source: J Ultrasound Med. 2025 Jul 25;44(12):2197–207. doi: 10.1002/jum.70001 (PMC12605691; doi:10.1002/jum.70001)
Supplement: Supplementary file 1 — Data S1. Supporting Information. [file JUM-44-2197-s001.pdf]

Supplementary material for "Sample size of trials investigating the impact of Point-Of-Care ultrasound guided strategies on patient outcomes: A systematic review"

1. Search strategy

Pubmed term: ((randomized controlled trial[pt] OR controlled clinical trial[pt] OR randomized[tiab] OR placebo[tiab] OR clinical trials as topic[mesh:noexp] OR randomly[tiab] OR trial[ti] NOT (animals[mh] NOT humans [mh]))) AND ((("point of care" OR point-of-care OR portable OR bedside OR bed-side OR handheld OR hand-held OR hand-carried OR pocket OR mobile) AND (ultrasoun* OR ultrason* OR sonogr* OR echo*)))

2. Scripts used for generating sample size estimation

2.1. Sample size for trials with a binary outcome

```
install.packages("rpact")
library(rpact)

# Load rpact
library(rpact)

# Define parameters
alpha <- 0.05
power <- 0.80
event_rate_control <- 0.40
effect_sizes <- c(0.60, 0.70, 0.80)  # OR values for intervention effect sizes

# Define adherence rates (rate of change in management in response to POCUS
information)
adherence_rates <- c(1.0, 0.75, 0.50, 0.25, 0.10)

# Prepare a list to store results
results <- list()

# Loop over each effect size
for (OR_full in effect_sizes) {
  # Calculate the adjusted event rates and ORs for each adherence level
  adjusted_event_rates <- event_rate_control / (1 + (1 / OR_full - 1) * adherence_rates)
  ORs_adjusted <- 1 - (1 - OR_full) * adherence_rates

  # Compute sample sizes for each adherence rate
  sample_sizes <- sapply(adjusted_event_rates, function(pi2) {
```

```
    design <- getDesignGroupSequential(kMax = 1, alpha = alpha, beta = 1 - power)
    sample_size <- getSampleSizeRates(
      design = design,
      groups = 2,
      pi1 = event_rate_control, # Control group rate
      pi2 = pi2,              # Intervention group rate adjusted for adherence
      allocationRatioPlanned = 1
    )
    sample_size$nFixed
  })

  # Store the results in a data frame for each effect size
  results[[as.character(OR_full)]] <- data.frame(
    EffectSize_OR = OR_full,
    Adherence = adherence_rates * 100,
    OR_Adjusted = ORs_adjusted,
    EventRate_Intervention = adjusted_event_rates,
    SampleSize = sample_sizes
  )
}

# Combine results into one data frame
final_result <- do.call(rbind, results)
print(final_result)
```

2.2. Sample size for trials with a continuous outcome

```
# Load rpact
library(rpact)

# Define parameters
alpha <- 0.05  # Significance level
sided <- 2     # Two-sided test
SD_fraction <- 0.8
mean_LOS <- 1
SD <- mean_LOS * SD_fraction
effect_sizes <- c(0.1, 0.2, 0.3, 0.4, 0.5)
adherence_rates <- c(1.0, 0.75, 0.5, 0.25, 0.1)

# Create a data frame to store results
results <- data.frame()

# Loop through combinations
```

```
for (effect_size in effect_sizes) {
 for (adherence in adherence_rates) {
  adjusted_effect_size <- effect_size * adherence
  design <- getDesignGroupSequential(kMax = 1, alpha = alpha, sided = sided)
  sample_size <- getSampleSizeMeans(
   design = design,
   alternative = adjusted_effect_size,
   stDev = SD
  )

  # Corrected extraction of sample size
  total_sample_size <- sample_size$maxNumberOfSubjects

  # Store results
  results <- rbind(results, data.frame(
   EffectSize = effect_size,
   Adherence = adherence * 100,
   AdjustedEffectSize = adjusted_effect_size,
   SampleSize = total_sample_size
  ))
 }
}

# View results
print(results)
```
